# Nutritional status, inflammatory burden, and CT-derived muscle phenotypes for predicting chronic postsurgical pain after total hip arthroplasty: an explainable machine learning study

**DOI:** 10.3389/fnut.2026.1958865

**Published:** 2026-10-20

**Authors:** Xiaodong Jia, Xinkun Han, Xinyu Cui, Yun Zhao, Yingze Zhang, Kuishuai Xu

**Affiliations:** 1 Knee Preservation Center, The Affiliated Hospital of Qingdao University, Qingdao, Shandong, China; 2 Department of Sports Medicine, The Affiliated Hospital of Qingdao University, Qingdao, Shandong, China; 3 School of Mathematical Sciences University of Electronic Science and Technology of China, Chengdu, China

**Keywords:** chronic postsurgical pain, machine learning, muscle parameters, nutritional status, total hip arthroplasty

## Abstract

**Background:**

Hip fracture (HF) is a common fragility fracture in older adults and may lead to persistent pain and impaired recovery. Chronic postsurgical pain (CPSP) after total hip arthroplasty (THA) negatively affects rehabilitation and quality of life. This study aimed to develop and validate a machine learning model integrating clinical variables, peri-hip muscle characteristics, and nutrition-related inflammatory indicators for CPSP prediction after THA in patients with HF.

**Methods:**

This single-center cohort study included patients with HF undergoing primary unilateral THA between October 2022 and March 2026. Patients from October 2022 to September 2024 were randomly divided into training and internal validation sets, while those from October 2024 to March 2026 served as an independent temporal test set. CPSP was defined as surgery-related pain persisting for ≥3 months with NRS ≥4. Candidate predictors included clinical, psychological, nutritional, inflammatory, and CT-derived muscle parameters. LASSO and Boruta were used for feature selection, and six machine learning algorithms were compared. SHAP analysis was applied to interpret the optimal model.

**Results:**

Among 881 patients, 137/529 (25.9%) developed CPSP in the development cohort. Seven predictors were selected: GNRI, gluteus medius/minimus muscle density, gluteus medius/minimus cross-sectional area, NLR, age, preoperative anxiety, and PNI. The SVM model showed the best performance, achieving an AUC of 0.854 in the temporal test set, with accuracy of 0.770, sensitivity of 0.789, specificity of 0.763, NPV of 0.913, F1-score of 0.637, and Brier score of 0.125. SHAP analysis identified GNRI as the most influential predictor (35.4%), followed by muscle density (13.6%) and NLR (12.5%).

**Conclusions:**

A machine learning model integrating clinical, CT-derived muscle, and nutrition-related inflammatory features showed good predictive performance for CPSP after THA in patients with HF and may support early risk stratification and individualized perioperative management.

## 1 Introduction

Hip fracture (HF) is one of the most common fragility fractures among older adults and represents a substantial global health burden in the context of population aging (1). With continued global population aging, the incidence of HF is expected to increase substantially over the coming decades, resulting in greater functional impairment and healthcare demands (1, 2). HF is associated not only with acute pain and mobility limitations but also with long-term functional decline, reduced quality of life, and increased mortality risk (3, 4). Total hip arthroplasty (THA) is an established treatment option for selected patients with hip fracture, providing favorable functional outcomes and contributing to postoperative recovery of mobility and quality of life (5, 6). However, despite advances in surgical management, a proportion of patients continue to experience suboptimal recovery, and chronic postsurgical pain (CPSP) has emerged as an important clinical concern affecting postoperative rehabilitation (7). CPSP is generally defined as pain that persists for at least 3 months after surgery and cannot be better explained by other causes, such as infection, implant-related complications, or recurrent trauma (7, 8). Persistent pain following HF surgery may compromise rehabilitation, reduce independence in daily activities, and negatively affect overall quality of life. Furthermore, prolonged pain may increase healthcare utilization and analgesic requirements while delaying functional recovery (9).

Skeletal muscle quality reflects the extent of intramuscular fat infiltration as well as metabolic and contractile characteristics. Computed tomography (CT) enables quantitative assessment of peri-hip skeletal muscle status using imaging-derived parameters. In addition to muscle area, CT can evaluate muscle quality by measuring Hounsfield units (HU), with lower HU values generally indicating greater intramuscular fat infiltration and poorer muscle quality (10). Previous studies have demonstrated that cross-sectional area (CSA) and HU are associated with postoperative mortality, physical function, walking ability, falls, and impaired functional recovery (11–14). Meanwhile, substantial evidence indicates that malnutrition is highly prevalent among older patients with hip fractures and is consistently associated with adverse clinical outcomes, including increased postoperative complications, prolonged hospital stays, delayed mobilization, and higher in-hospital and mortality rates (15, 16). Furthermore, emerging evidence suggests that nutritional deficits and systemic inflammation may modulate pain perception and recovery trajectories, linking metabolic status to both acute and chronic postoperative pain (17, 18). Several routinely available laboratory-based indicators, including the Geriatric Nutritional Risk Index (GNRI), Prognostic Nutritional Index (PNI), and Systemic Immune-Inflammation Index (SII), have been demonstrated to be associated with adverse outcomes in older patients with hip fractures (19, 20). These biomarkers objectively reflect nutritional reserve, immune function, and inflammatory burden, and may provide valuable information for risk stratification. However, the association between these commonly used clinical indicators and chronic postoperative pain after hip fracture surgery remains unclear. Despite these findings, whether peri-hip muscle quality and nutrition-related inflammatory indicators influence chronic postoperative pain remains unknown.

Machine learning (ML), as an approach capable of effectively handling complex nonlinear relationships and high-dimensional data, has been widely applied in clinical prediction models and may facilitate clinical decision-making. Therefore, we aimed to develop and validate a ML model that integrates routinely available clinical variables, peri-hip muscle quality, and nutrition-related inflammatory indicators to predict the risk of CPSP after THA in older patients with HF, thereby providing a basis for early identification, risk stratification, and individualized interventions in high-risk patients.

## 2 Methods

### 2.1 Study design and participants

This study employed a single-center cohort design and aimed to develop and validate a machine learning-based prediction model for predicting the risk of chronic postsurgical pain after THA in patients with HF. Consecutive patients with HF who underwent primary unilateral THA at the Affiliated Hospital of Qingdao University between October 2022 and March 2026 were included. The inclusion criteria were as follows: (1) adult patients aged ≥50 years; (2) patients who underwent primary unilateral THA due to HF; (3) availability of complete preoperative clinical data, including demographic characteristics, comorbidities, preoperative pain status, psychological-related indicators, and perioperative information; (4) availability of preoperative CT images allowing measurement of muscle imaging parameters, including muscle cross-sectional area and muscle density; and (5) availability of complete preoperative laboratory test results for calculating nutrition- and inflammation-related indicators. The exclusion criteria were as follows: (1) previous ipsilateral hip arthroplasty or a history of major surgery affecting functional evaluation of the affected hip; (2) concomitant malignancy, active infection, or severe systemic diseases that could affect postoperative pain recovery or clinical outcome assessment; (3) severe cognitive impairment, psychiatric disorders, or inability to complete pain and psychological assessments; (4) unavailable or inadequate preoperative CT images that prevented muscle parameter measurements; and (5) missing key clinical variables or follow-up data. According to the chronological order of patient enrollment, the study cohort was divided into a model development phase and an independent temporal validation phase. Patients consecutively enrolled between October 2022 and September 2024 were used for model development and were further divided into a training set and an internal validation set. The training set was used for ML model development, feature selection, and parameter optimization, whereas the internal validation set was used to compare the predictive performance of different models and determine the optimal prediction model. Subsequently, patients consecutively enrolled between October 2024 and March 2026 were included as an independent temporal test set to evaluate the predictive performance, stability, and generalizability of the final model in patients who were not involved in model development (Figure 1). The study protocol was reviewed and approved by the Ethics Committee of the Affiliated Hospital of Qingdao University (approval number: QYFY WZLL 42077). This study was conducted in accordance with the ethical principles of the Declaration of Helsinki. All study data were anonymized to ensure patient privacy and information security and were used exclusively for data analysis in the present study.

**Figure 1:**
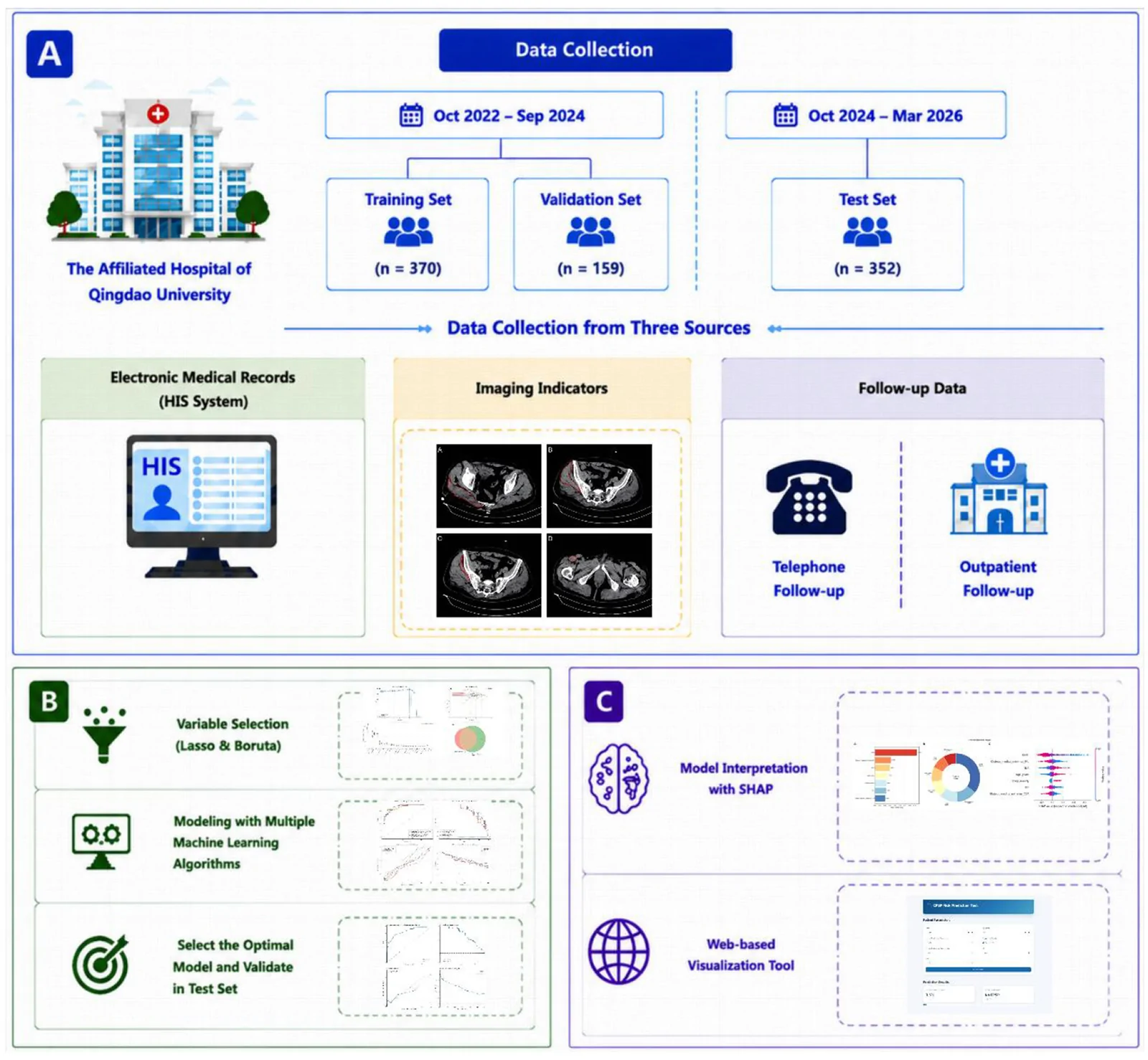
Flow diagram of patient selection and study design. The flowchart illustrates the patient inclusion, exclusion, cohort allocation, and follow-up processes in patients with HF undergoing THA. Eligible patients were assigned to the development cohort for machine learning model construction and internal validation, and an independent temporal validation cohort was used to evaluate the generalizability of the final model. **(A)** Data collection at the Affiliated Hospital of Qingdao University from October 2022 to March 2026, with allocation into training, internal validation, and temporal test sets. Data sources included electronic medical records, imaging-derived indicators, and outpatient or telephone follow-up. **(B)** Workflow of variable selection, machine learning model development, and model validation. **(C)** SHAP-based model interpretation and a web-based visualization tool.

### 2.2 Definition of the primary outcome

The primary outcome of this study was the occurrence of CPSP after THA in patients with HF. Outcome assessment was conducted by trained members of the research team using the standardized NRS and prespecified CPSP criteria. Demographic characteristics of the outcome assessors were not collected. All patients were followed up for at least 3 months after surgery, and postoperative pain-related information was collected through outpatient visits or telephone interviews. According to the definition of CPSP proposed by the International Association for the Study of Pain (IASP), CPSP was defined as pain that newly developed after surgery or an increase in pre-existing pain intensity and persisted for at least 3 months (7). Surgery-related pain was defined as pain that newly developed after surgery or increased compared with the preoperative level and was located in or around the operated hip. CPSP was adjudicated based on follow-up symptoms, clinical records, and available imaging findings, after excluding infection, implant loosening or dislocation, periprosthetic fracture, recurrent trauma, referred pain, and other identifiable musculoskeletal causes. Outcome assessors were unaware of the candidate predictors used for model development when determining CPSP status. Postoperative pain was assessed using the numeric rating scale (NRS), with scores ranging from 0 to 10, where 0 indicates no pain and 10 represents the most severe pain imaginable. In this study, pain status at the 3-month postoperative follow-up was used as the primary outcome assessment. Specifically, patients who continued to experience surgery-related pain with an NRS score ≥4 at 3 months after surgery were classified into the CPSP group, whereas patients without persistent pain or with an NRS score < 4 were classified into the non-CPSP group.

### 2.3 Collection and definition of candidate predictors

Based on previous literature, clinical experience, and potential mechanisms associated with CPSP development, candidate predictors were prespecified before model development. These variables mainly included demographic characteristics, comorbidities, perioperative information, pain-related factors, psychological factors, nutrition- and inflammation-related indicators, and imaging-derived muscle parameters. Demographic characteristics and baseline clinical information were collected, including age, sex, body mass index, education level (categorized as ≥secondary education or < secondary education according to the highest educational attainment), American Society of Anesthesiologists (ASA) classification, and comorbidities such as hypertension, diabetes mellitus, and cardiovascular diseases. Perioperative variables, including anesthesia type, operative time, intraoperative blood loss, blood transfusion status, and postoperative length of hospital stay, were also recorded. Pain-related factors included preoperative pain intensity, pain intensity at 48 h after surgery, and a history of long-term analgesic use before surgery. Preoperative long-term analgesic use was defined as regular analgesic medication use for at least 3 months before admission. Pain severity was assessed using the NRS. Psychological factors included preoperative anxiety, depression, and pain catastrophizing. Anxiety and depression were evaluated using the Hospital Anxiety and Depression Scale, whereas pain catastrophizing was assessed using the Short-Form Pain Catastrophizing Scale (SF-PCS) (21, 22). These assessments were used to evaluate the potential influence of preoperative psychological status and pain-related cognitive characteristics on the development of CPSP. The validated Chinese versions of both instruments were used. Scores of ≥8 on the HADS-A and HADS-D subscales were used to define anxiety and depression, respectively. The SF-PCS comprised seven items, with a total score of ≥7 indicating clinically significant pain catastrophizing (23, 24). Nutrition- and inflammation-related indicators were obtained from routine preoperative laboratory tests, including serum albumin, GNRI, PNI, neutrophil-to-lymphocyte ratio (NLR), C-reactive protein-to-albumin ratio (CAR), and SII. These indicators were used to comprehensively evaluate patients' preoperative nutritional status, inflammatory burden, and physiological reserve. The calculation formulas of these indicators are as follows: GNRI = 1.489 × Albumin + 41.7 × (Actual weight/Ideal weight); PNI = Albumin + 5 × Lymphocyte; NLR = Neutrophil/Lymphocyte; CAR = (C-reactive protein)/Albumin; SII = (Platelet × Neutrophil)/Lymphocyte. Preoperative CT images were used to assess skeletal muscle status. Based on previous studies of opportunistic CT-based muscle assessment and hip functional recovery, muscle groups closely associated with lower-limb function and systemic muscle reserve were selected for analysis, including the gluteus maximus, gluteus medius/minimus, and rectus femoris muscles. The CSA and muscle density measured by HU were obtained through standardized CT image analysis, reflecting muscle quantity and muscle quality, respectively. All candidate predictors were organized before model development and subsequently used for feature selection, model training, and evaluation of predictive performance in the ML models.

### 2.4 Measurement of imaging-derived muscle parameters

All patients underwent preoperative CT examination, and the imaging data were used to evaluate preoperative skeletal muscle status. Considering that the fractured side of the hip may exhibit alterations in muscle morphology due to pain, immobilization, and local injury, the contralateral lower limb and peri-hip muscles were selected for measurement to more accurately reflect the patients' overall preoperative muscle reserve. Based on previous studies of opportunistic CT-based muscle assessment and postoperative recovery in older patients with HF, muscle groups closely associated with hip stability, lower-limb function, and systemic muscle reserve were selected for analysis, including the gluteus maximus, gluteus medius/minimus, and rectus femoris muscles (25–27). The specific measurement locations were as follows: the gluteus maximus was measured at the level of the acetabular roof; the gluteus medius/minimus was measured at the level inferior to the sacroiliac joint; and the rectus femoris was measured below the lesser trochanter (Figure 2). Preoperative CT imaging data were imported into imaging analysis software in a standard format. Trained researchers manually delineated the boundaries of the target muscles at the corresponding anatomical levels and measured their CSA and mean CT attenuation values. Muscle CSA was used to reflect muscle quantity, whereas mean CT attenuation values were used to reflect muscle quality and the extent of intramuscular fat infiltration. All muscle parameters were analyzed using measurements obtained from the contralateral side. To ensure measurement reliability, all imaging assessments were performed by researchers who received standardized training. The assessors were blinded to postoperative pain outcomes during image evaluation to minimize observer bias. A randomly selected subset of 20 patients underwent repeated measurements by a second researcher, and the consistency of measurements was evaluated using the intraclass correlation coefficient (ICC).

**Figure 2:**
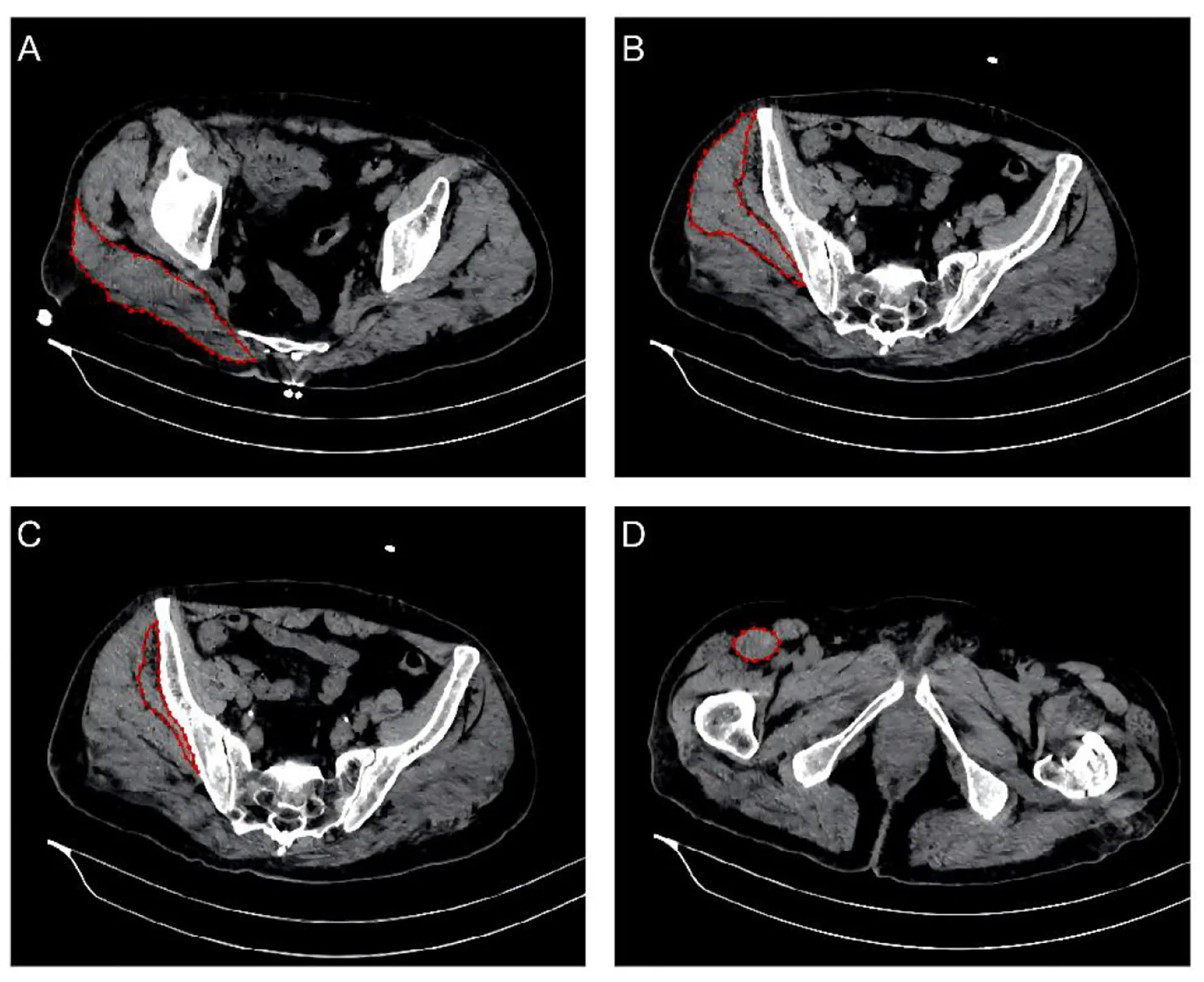
CT-derived measurement of peri-hip skeletal muscle parameters. Representative axial CT images demonstrating the measurement of peri-hip skeletal muscle parameters. The cross-sectional area (CSA) and muscle density (HU) of the gluteus maximus, gluteus medius/minimus, and rectus femoris were assessed at different anatomical levels. **(A)** The gluteus maximus was measured at the level of the acetabular roof, **(B, C)** the gluteus medius/minimus was measured below the sacroiliac joint, and **(D)** the rectus femoris was measured below the lesser trochanter. Regions of interest were manually delineated to obtain CSA and mean HU values, representing muscle quantity and quality, respectively.

### 2.5 Development and validation of ML models

ML approaches were used to develop and validate prediction models for CPSP after THA in patients with HF. The model development process consisted of data preprocessing, feature selection, model training, hyperparameter optimization, internal validation, independent temporal testing, and model interpretation. Before model development, all candidate predictors underwent data preprocessing. No oversampling, undersampling, or SMOTE procedure was applied. Class weighting was evaluated as a candidate setting for selected algorithms, but the final SVM model did not use class weighting. Continuous variables were standardized based on the training set to reduce the influence of different measurement scales on model training, whereas categorical variables were transformed using appropriate encoding methods. Before model development, data completeness was assessed for all candidate predictors and the primary outcome. All patients included in the final analytic cohort had complete data for all candidate predictors, CT-derived muscle measurements, and the primary outcome; therefore, no missing-data imputation was performed. All preprocessing procedures were performed exclusively using the training set and subsequently applied to the internal validation set and independent temporal test set to prevent data leakage. Within the training set, two complementary feature selection approaches were applied to identify relevant predictors: least absolute shrinkage and selection operator (LASSO) regression and the Boruta algorithm. LASSO performs variable selection by introducing a penalty term to shrink regression coefficients and reduce the risk of overfitting. The Boruta algorithm, based on the random forest framework, identifies stable predictive features by comparing the importance of original variables with randomly permuted variables. To improve feature selection robustness, the intersection of variables selected by LASSO and Boruta was used as the final predictor set, retaining only variables consistently identified as having predictive value by both approaches. Feature selection was not repeated within each cross-validation training fold, and neither the internal validation set nor the independent temporal test set was used for feature selection or hyperparameter optimization. Based on the selected predictors, multiple ML algorithms were developed, including logistic regression, random forest (RF), support vector machine (SVM), extreme gradient boosting (XGBoost), categorical Boosting (CatBoost), and k-nearest neighbors (KNN). During model training, cross-validation was performed for hyperparameter optimization. The predictive performance of different algorithms was compared in the training and internal validation sets, and the optimal model was selected based on comprehensive performance evaluation. The final selected model was subsequently applied to the independent temporal test set to evaluate its predictive performance, stability, and generalizability in patients who were not involved in model development. Model performance was assessed from three aspects: discrimination, calibration, and clinical utility. Discrimination was evaluated using receiver operating characteristic (ROC) curves and the area under the curve (AUC). The classification threshold was determined by maximizing the Youden index based on five-fold out-of-fold predictions from the training set and was subsequently applied unchanged to the internal validation and independent temporal test sets. Calibration was assessed using calibration curves and the Brier score to evaluate the agreement between predicted probabilities and observed outcomes. Decision curve analysis (DCA) was performed to determine the clinical net benefit of the model across different risk thresholds. To enhance the interpretability of the optimal model, Shapley additive explanations (SHAP) analysis was performed to explain model predictions. The contribution of each predictor to model output was quantified to determine the importance of individual variables for CPSP risk prediction and to illustrate the direction of their effects on individual risk estimation. SHAP values were calculated for all patients in the training set using the model-agnostic permutation explainer implemented in the Python SHAP package. Sixty randomly selected observations from the training set were used as the background dataset, and the predicted probability of CPSP was used as the model output. Furthermore, an individualized risk prediction tool was developed based on the final optimal model. By incorporating patients' clinical characteristics, pain-related factors, nutrition- and inflammation-related indicators, and CT-derived muscle parameters, the tool enables individualized prediction of CPSP risk and may assist perioperative risk stratification and personalized management for patients with HF undergoing THA.

### 2.6 Statistical analysis

All statistical analyses were performed using Python software (version 3.10). Normality was assessed separately for the groups included in each comparison. Continuous variables were presented as mean ± standard deviation and compared using the independent-samples *t*-test when both groups were approximately normally distributed. When either group showed a non-normal distribution, variables were presented as median (interquartile range) and compared using the Mann–Whitney U test. In the development cohort (n = 529), a total of 137 CPSP events were observed. Based on the 137 CPSP events and seven final selected predictors, the events-per-variable ratio was 19.57. This event-to-predictor ratio was considered adequate for model development, although it does not completely exclude the possibility of overfitting. This study was reported in accordance with the Transparent Reporting of a Multivariable Prediction Model for Individual Prognosis or Diagnosis Plus Artificial Intelligence (TRIPOD+AI) guideline. All statistical tests were two-sided, and *P* < 0.05 was considered statistically significant.

## 3 Results

### 3.1 Patient enrollment, dataset allocation, and baseline characteristics

A total of 881 patients with HF undergoing THA were included in this study. According to the chronological order of enrollment, 529 patients enrolled between October 2022 and September 2024 were assigned to the development cohort, which was randomly divided into a training set (*n* = 370) and an internal validation set (*n* = 159). Additionally, 352 patients enrolled between October 2024 and March 2026 were included as an independent temporal test cohort (Figure 1). Among patients in the development cohort, 137 patients developed CPSP, with an incidence rate of 25.9%. Baseline characteristics according to CPSP status are summarized in Table 1. Compared with patients without CPSP, patients with CPSP were older and had higher proportions of preoperative long-term analgesic use, smoking history, diabetes mellitus, preoperative anxiety, and pain catastrophizing (all *P* < 0.05). Patients with CPSP also demonstrated poorer peri-hip muscle status, characterized by lower gluteus maximus HU, reduced gluteus medius/minimus CSA, and lower gluteus medius/minimus HU (all *P* < 0.05). Furthermore, CPSP patients exhibited greater nutritional impairment and inflammatory burden, including lower GNRI and PNI, and higher CAR, NLR, and SII levels (all P < 0.05). To evaluate the comparability between datasets, baseline characteristics between the development cohort and independent temporal test cohort were further compared (Supplementary Table 1). No significant differences were observed across all evaluated variables, including demographic characteristics, pain-related factors, psychological factors, CT-derived muscle parameters, perioperative variables, and nutrition- and inflammation-related indicators (all *P* > 0.05). The incidence of CPSP was also comparable between the two cohorts (137/529 vs 90/352, *P* = 0.913), supporting the suitability of the temporal test cohort for model validation. The intra- and inter-observer reliability for all measured muscle parameters was excellent (Supplementary Table 2). Intra-observer ICCs ranged from 0.865 to 0.983, with rectus femoris CSA showing the highest agreement (ICC = 0.983, 95% CI: 0.958–0.993). Inter-observer ICCs ranged from 0.850 to 0.966, led again by rectus femoris CSA (ICC = 0.966, 95% CI: 0.918–0.986). Since all ICC values exceeded 0.75, all six parameters demonstrated high reproducibility and were included in further analyses.

**Table 1:** Comparison of baseline characteristics and candidate predictors between patients with and without chronic postsurgical pain in the development cohort.

Variable	CPSP (*n* = 137)	No CPSP (*n* = 392)	*P*-value
Preoperative NRS (score)	7.00 (5.00, 8.00)	7.00 (6.00, 7.00)	0.800
Postoperative 48-h NRS (score)	4.00 (3.00, 5.00)	4.00 (3.00, 4.00)	0.104
Age (years)	69.99 ± 5.82	66.19 ± 5.88	< 0.001
Sex (Male/Female)	51/86	139/253	0.711
BMI (kg/m2)	23.27 (17.60, 31.55)	23.91 (21.36, 26.59)	0.497
Education level (≥Secondary/ < Secondary)	51/86	158/234	0.526
Preoperative long-term analgesic use (Yes/No)	63/74	110/282	< 0.001
Smoking history (Yes/No)	68/69	126/266	< 0.001
Alcohol history (Yes/No)	37/100	127/265	0.240
Hypertension (Yes/No)	72/65	177/215	0.135
Diabetes (Yes/No)	60/77	67/325	< 0.001
Heart disease (Yes/No)	38/99	94/298	0.382
History of cerebral infarction (Yes/No)	26/111	76/316	0.917
Preoperative anxiety (Yes/No)	73/64	75/317	< 0.001
Preoperative depression (Yes/No)	29/108	71/321	0.432
Pain catastrophizing (Yes/No)	53/84	76/316	< 0.001
Gluteus maximus CSA (cm2)	30.31 ± 6.96	30.49 ± 7.02	0.791
Gluteus maximus HU (HU)	21.82 (19.60, 26.76)	24.05 (20.33, 29.47)	0.002
Rectus femoris CSA (cm2)	3.69 (2.87, 4.57)	3.76 (2.72, 4.73)	0.384
Rectus femoris HU (HU)	40.95 (31.64, 50.18)	41.38 (38.27, 45.14)	0.700
Gluteus medius/minimus CSA (cm2)	27.70 (23.02, 32.13)	31.54 (26.85, 36.95)	< 0.001
Gluteus medius/minimus HU (HU)	28.27 (25.01, 31.85)	32.88 (28.84, 37.24)	< 0.001
ASA physical status (III–IV/I–II)	67/70	184/208	0.692
General anesthesia (Yes/No)	88/49	270/122	0.317
Surgery delay >48 h (Yes/No)	91/46	248/144	0.507
High-energy injury (Yes/No)	43/94	103/289	0.249
Surgery duration (min)	60.00 (50.00, 85.00)	70.00 (60.00, 70.00)	0.159
Operative side (Right/Left)	59/78	203/189	0.079
Intraoperative blood loss (mL)	390.00 (310.00, 490.00)	410.00 (360.00, 430.00)	0.775
Transfusion (Yes/No)	34/103	92/300	0.750
CAR	1.73 (1.18, 2.20)	1.49 (1.00, 2.05)	0.002
GNRI	88.19 ± 3.04	93.84 ± 2.86	< 0.001
PNI	40.35 (37.50, 42.65)	42.15 (39.70, 45.00)	< 0.001
NLR	8.15 (6.47, 10.23)	6.30 (4.32, 8.16)	< 0.001
SII	1,192.92 (777.05, 2,277.79)	1,019.50 (654.98, 1,619.14)	0.017

NRS, Numeric Rating Scale; CPSP, chronic postsurgical pain; BMI, body mass index; CSA, cross-sectional area; HU, Hounsfield units; ASA, American Society of Anesthesiologists; CAR, C-reactive protein-to-albumin ratio; GNRI, Geriatric Nutritional Risk Index; PNI, Prognostic Nutritional Index; NLR, neutrophil-to-lymphocyte ratio; SII, Systemic Immune-Inflammation Index.

### 3.2 Feature selection and identification of final predictors

To identify stable predictors and reduce the risk of model overfitting, LASSO regression combined with the Boruta algorithm was applied for feature selection. LASSO regression demonstrated that as the regularization parameter increased, the coefficients of some variables gradually shrank toward zero, whereas only selected variables retained relatively higher contributions (Figure 3A). The LASSO coefficient path further illustrated the changing contribution of individual variables across different regularization levels (Figure 3B). Subsequently, the Boruta algorithm based on the random forest framework was performed to evaluate variable importance and identify confirmed variables with stable predictive value (Figure 3C). The intersection analysis between LASSO-selected variables and Boruta-confirmed variables identified seven stable candidate predictors (Figure 3D). These predictors were subsequently used for ML model development and included GNRI, gluteus medius/minimus muscle density, gluteus medius/minimus CSA, preoperative anxiety, NLR, age, and PNI.

**Figure 3:**
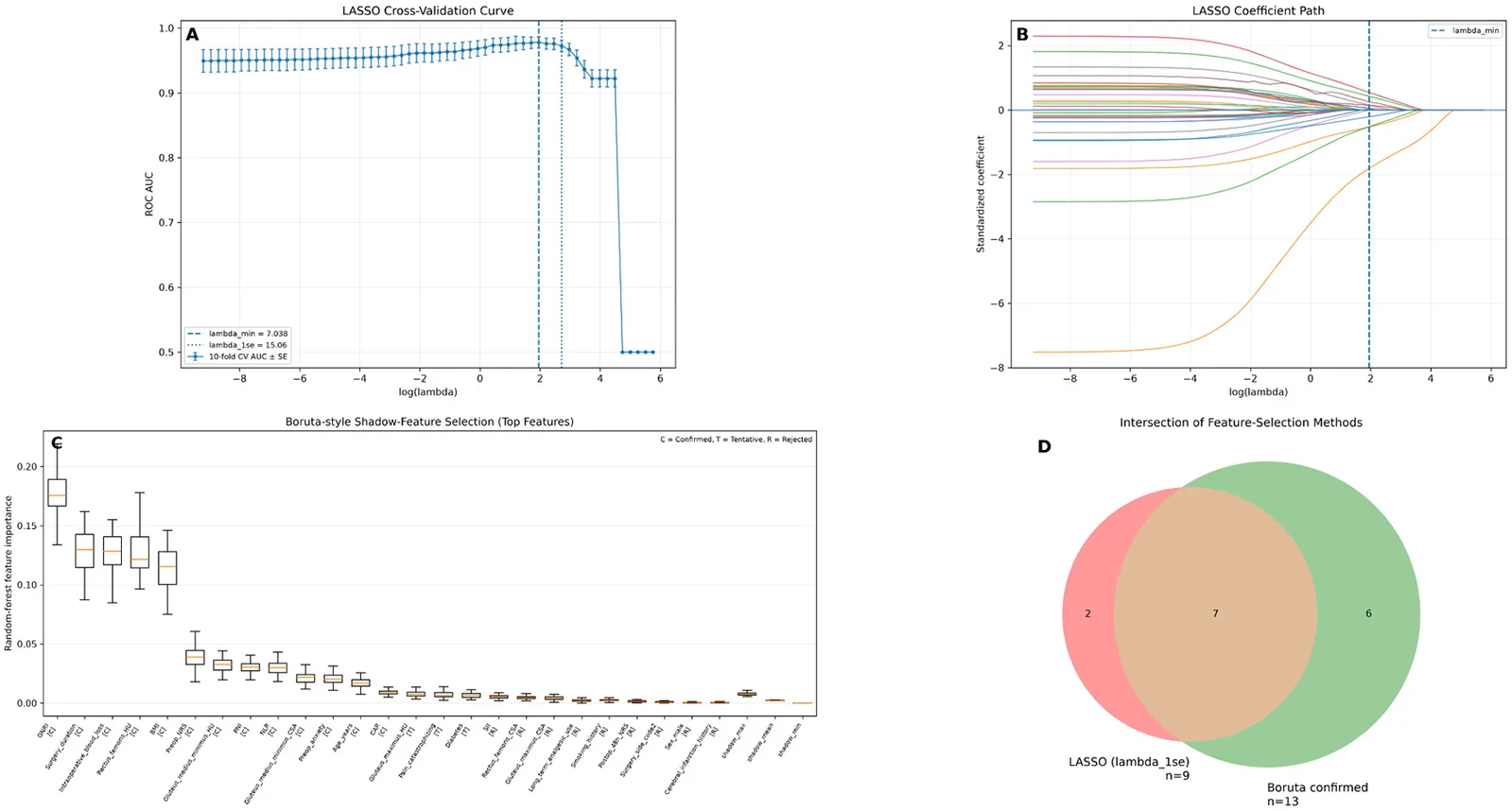
Feature selection using LASSO regression and the Boruta algorithm. **(A)** Cross-validation curve for determining the optimal penalty parameter in the LASSO model. **(B)** LASSO coefficient profiles showing variable selection during model development. **(C)** Variable importance ranking generated by the Boruta algorithm. **(D)** Overlapping predictors identified by LASSO and Boruta, representing the final features incorporated into the machine learning models.

### 3.3 Development and performance comparison of multiple ML models

Based on the selected predictors, six ML models, including logistic regression, RF, SVM, XGBoost, CatBoost, and KNN, were developed and compared for their predictive performance. In the internal validation set, all models demonstrated good predictive performance (Table 2). Among these models, logistic regression and SVM achieved the highest AUC values (both 0.944). The SVM model showed the highest sensitivity (0.886) and negative predictive value (0.949), indicating its favorable ability for CPSP risk discrimination. ROC curve analysis demonstrated good discrimination ability across all models, with logistic regression and SVM showing the best performance (Figure 4A). Precision–Recall curve analysis further indicated good classification performance of all models, with the logistic regression model achieving the highest AUPRC (0.897) (Figure 4B). Calibration curve analysis showed overall agreement between predicted probabilities and observed outcomes among all models (Figure 4C). Decision curve analysis demonstrated that all models provided clinical net benefit across certain threshold probability ranges, with the SVM model demonstrating relatively consistent net benefit across clinically relevant threshold probabilities (Figure 4D). Although several models showed comparable discrimination performance, SVM demonstrated superior sensitivity and negative predictive value, which may be clinically valuable for minimizing missed identification of patients at risk of CPSP. Considering model discrimination, sensitivity, and interpretability, the SVM model was ultimately selected as the optimal prediction model for subsequent analysis.

**Table 2:** Performance comparison of machine learning models in the training and internal validation cohorts and evaluation of model performance in the independent temporal test cohort.

Dataset	ML	AUC (95%CI)	ACC	Sensitivity	Specificity	PPV	NPV	F1-score	Brier
Training set	LR	0.974 (0.957–0.988)	0.916	0.903	0.921	0.792	0.966	0.844	0.069
	RF	0.963 (0.943–0.980)	0.908	0.871	0.921	0.786	0.955	0.827	0.073
	SVM	0.974 (0.956–0.988)	0.884	0.978	0.852	0.689	0.992	0.809	0.050
	XGBoost	0.966 (0.947–0.981)	0.903	0.882	0.910	0.766	0.958	0.820	0.064
	CatBoost	0.971 (0.954–0.985)	0.932	0.871	0.953	0.862	0.957	0.866	0.058
	KNN	0.955 (0.930–0.975)	0.865	0.903	0.852	0.672	0.963	0.771	0.069
Validation set	LR	0.944 (0.904–0.976)	0.868	0.705	0.930	0.795	0.892	0.747	0.088
	RF	0.907 (0.851–0.953)	0.855	0.636	0.939	0.800	0.871	0.709	0.108
	SVM	0.944 (0.905–0.976)	0.836	0.886	0.817	0.650	0.949	0.750	0.082
	XGBoost	0.919 (0.864–0.963)	0.855	0.727	0.904	0.744	0.897	0.736	0.098
	CatBoost	0.918 (0.864–0.963)	0.862	0.682	0.930	0.789	0.884	0.732	0.106
	KNN	0.920 (0.864–0.965)	0.836	0.818	0.843	0.667	0.924	0.735	0.099
Test set	SVM	0.854 (0.803–0.899)	0.770	0.789	0.763	0.534	0.913	0.637	0.125

ML, machine learning; AUC, area under the receiver operating characteristic curve; ACC, accuracy; PPV, positive predictive value; NPV, negative predictive value; LR, logistic regression; RF, random forest; SVM, support vector machine; XGBoost, Extreme Gradient Boosting; CatBoost, Categorical Boosting; KNN, k-nearest neighbors; CI, confidence interval.

**Figure 4:**
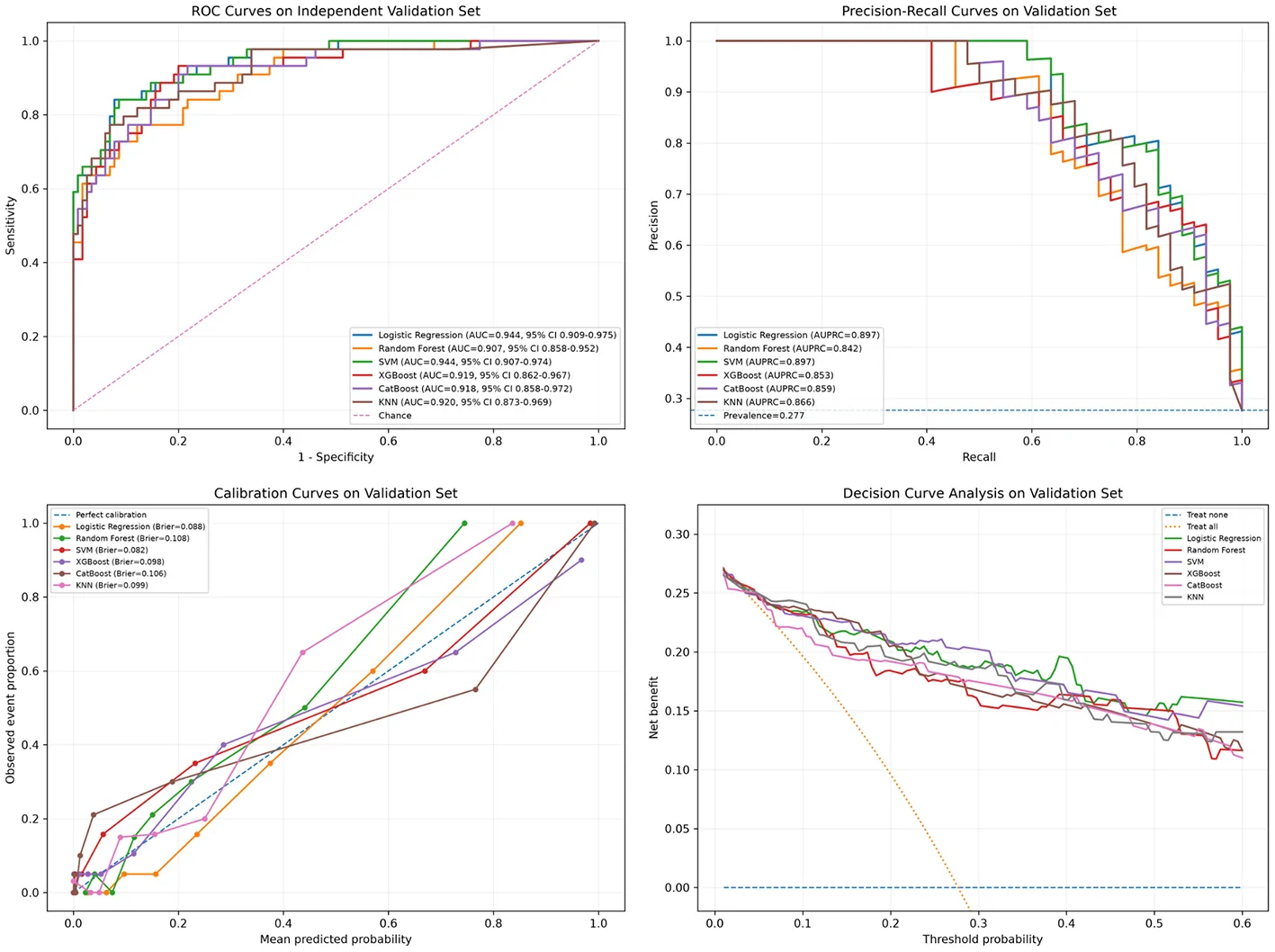
Performance evaluation of machine learning models in the internal validation cohort. **(A)** Receiver operating characteristic curves comparing the discrimination performance of six machine learning models for predicting chronic postsurgical pain after total hip arthroplasty. **(B)** Precision–recall curves evaluating the classification performance of different machine learning models, considering the imbalance between CPSP and non-CPSP groups. **(C)** Calibration curves assessing the agreement between predicted probabilities and observed CPSP outcomes. **(D)** Decision curve analysis evaluating the clinical net benefit of different machine learning models across a range of threshold probabilities.

### 3.4 Validation in the independent temporal test set

To further evaluate the generalizability of the model in an independent temporal cohort, the final SVM model was validated using the independent temporal test set (n=352). In this test set, the SVM model maintained stable predictive performance, with an AUC of 0.854, accuracy of 0.770, sensitivity of 0.789, specificity of 0.763, PPV of 0.534, NPV of 0.913, F1-score of 0.637, and Brier score of 0.125 (Table 2). ROC curve analysis demonstrated that the SVM model maintained good discrimination ability in patients who were not involved in model training, achieving an AUC of 0.854 and indicating favorable CPSP risk discrimination (Figure 5A). Precision–Recall curve analysis further showed an AUPRC of 0.754, which was higher than the random baseline level, suggesting that the SVM model retained effective identification ability for patients with CPSP despite the relatively low event prevalence (Figure 5B). Calibration analysis showed good agreement between predicted probabilities and observed outcomes, with a Brier score of 0.125, indicating stable probability estimation performance (Figure 5C). Decision curve analysis demonstrated that the SVM model achieved greater net benefit than the “treat all” and “treat none” strategies across certain risk threshold ranges, suggesting potential clinical utility of the model (Figure 5D).

**Figure 5:**
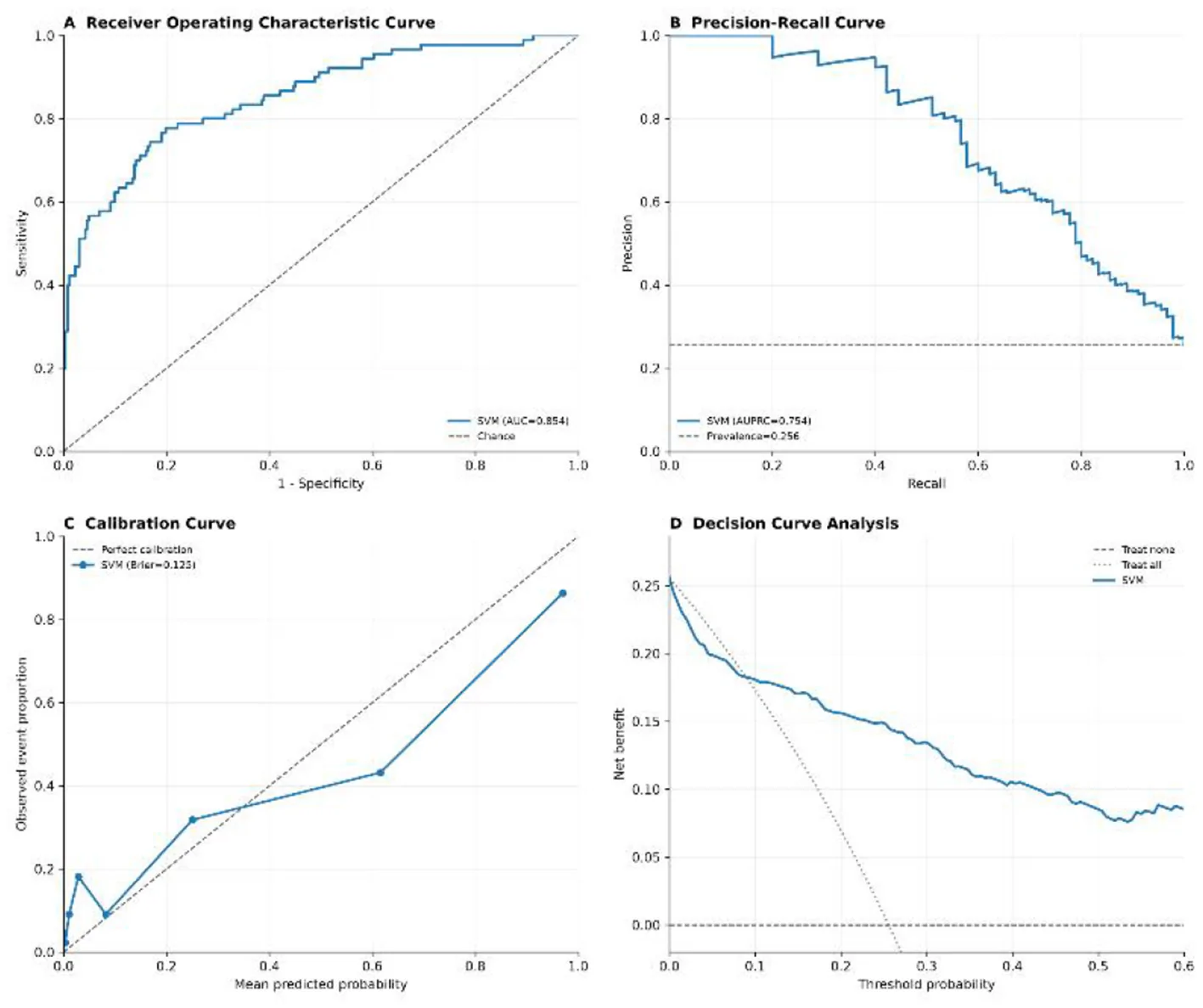
Performance evaluation of the final SVM model in the independent temporal validation cohort. **(A)** ROC curve demonstrating the discrimination ability of the SVM model. **(B)** Precision–recall curve assessing model performance in the temporal validation cohort. **(C)** Calibration curve evaluating the agreement between predicted and observed CPSP probabilities. **(D)** Decision curve analysis demonstrating the clinical utility of the SVM model across different threshold probabilities. In the independent temporal test cohort, logistic regression and SVM showed nearly identical discrimination, with an AUC of 0.854 for both models. Logistic regression achieved higher accuracy, specificity, and positive predictive value, whereas SVM achieved higher sensitivity and negative predictive value. Detailed comparative results are presented in Supplementary Table 3. Given the screening-oriented purpose of the study, SVM was retained as the primary model.

### 3.5 SHAP-based model interpretation

To improve the interpretability of the ML model, SHAP analysis was performed to quantify the contribution of each predictor to CPSP risk prediction in the SVM model. SHAP feature importance ranking showed that GNRI was the most influential predictor, followed by gluteus medius/minimus muscle density, NLR, age, preoperative anxiety, PNI, and gluteus medius/minimus CSA (Figure 6A). The feature contribution analysis demonstrated that GNRI accounted for the largest proportion of model contribution (35.4%), followed by gluteus medius/minimus muscle density (13.6%), NLR (12.5%), age (10.8%), preoperative anxiety (9.7%), PNI (9.5%), and gluteus medius/minimus CSA (8.4%) (Figure 6B). The SHAP summary plot further demonstrated that lower GNRI, lower gluteus medius/minimus muscle density, higher NLR, older age, and preoperative anxiety were associated with increased predicted CPSP risk, whereas lower PNI and lower muscle-related parameters showed an overall trend toward higher predicted risk (Figure 6C).

**Figure 6:**
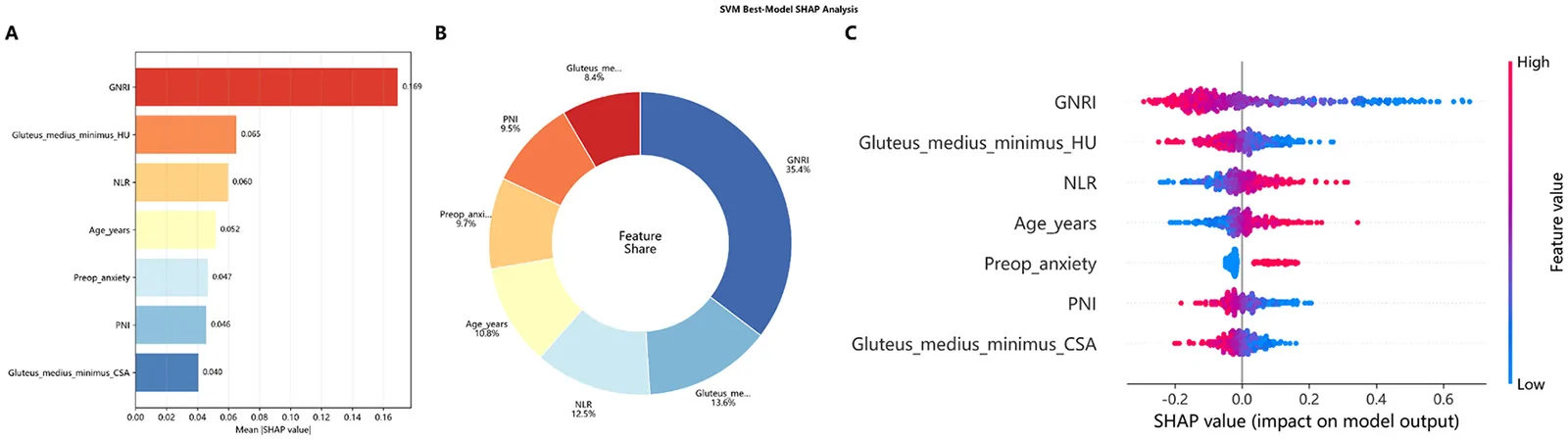
SHAP-based feature importance and model interpretability. **(A)** Global feature importance ranking based on mean absolute SHAP values. **(B)** Feature share representation illustrating the proportional contribution of key predictors to overall model interpretability. **(C)** SHAP summary plot showing the distribution and directional impact of feature values on CPSP risk.

### 3.6 Web-based individualized risk prediction tool

To facilitate clinical application of the developed model, a web-based risk prediction tool was established based on the final SVM model (Figure 7). The online calculator is publicly accessible at https://cpsp-risk-predictor-ml12.pages.dev/. The tool integrates the seven selected predictors, including GNRI, gluteus medius/minimus muscle density, gluteus medius/minimus CSA, preoperative anxiety, NLR, age, and PNI, allowing individualized estimation of CPSP risk after THA in patients with HF. By entering patient-specific predictor values available before hospital discharge, the tool automatically generates the predicted probability of CPSP and provides an individualized estimate of CPSP risk. This web-based tool provides a convenient approach for translating the machine-learning model into clinical practice and may assist clinicians in identifying patients at increased risk of CPSP before hospital discharge.

**Figure 7:**
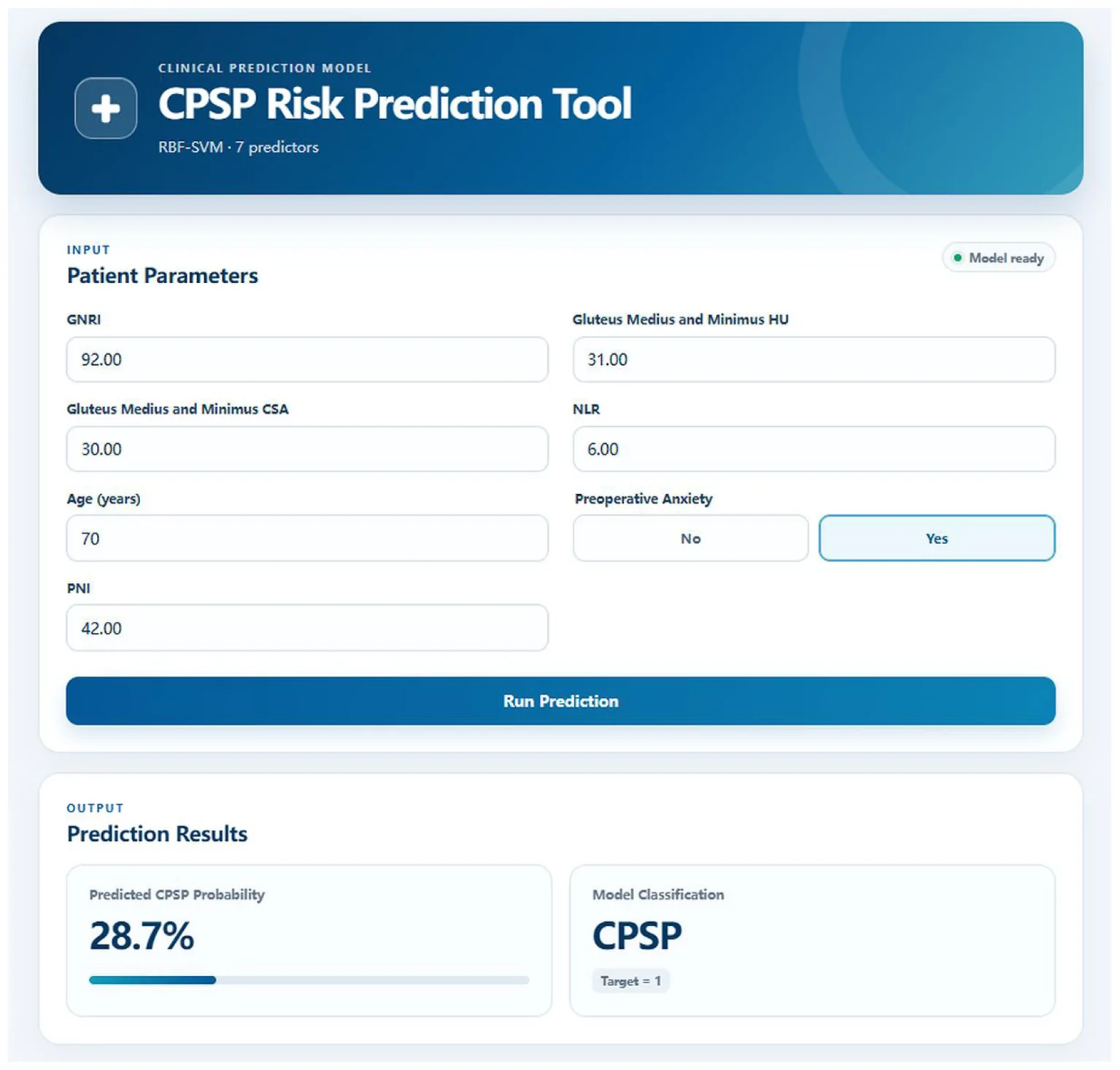
Web-based interface for individualized CPSP risk prediction. The web-based application developed from the final SVM model integrates seven selected predictors, including GNRI, gluteus medius/minimus muscle density, gluteus medius/minimus CSA, preoperative anxiety, NLR, age, and PNI. The interface automatically estimates the probability of CPSP based on patient-specific preoperative characteristics.

## 4 Discussion

The present study developed and validated a machine learning-based multidimensional prediction model to estimate the risk of CPSP after THA in patients with HF. Unlike previous risk assessment approaches that primarily relied on conventional factors such as age, preoperative pain intensity, and psychological characteristics, this study further incorporated preoperative nutritional status, inflammatory burden, psychological profiles, and CT-derived muscle phenotypes to explore potential determinants of CPSP from the perspective of overall recovery capacity. Through the combination of LASSO regression and the Boruta algorithm, seven key predictors were identified, including GNRI, gluteus medius/minimus muscle density, gluteus medius/minimus CSA, preoperative anxiety, NLR, age, and PNI. The independent temporal test demonstrated that the SVM model established based on these features maintained favorable predictive performance, suggesting that CPSP development may not be determined solely by surgical trauma and the intensity of nociceptive stimulation. Instead, CPSP appears to result from the interaction of multiple systemic factors, including preoperative physiological reserve, inflammatory status, psychological susceptibility, and functional capacity.

The present study identified GNRI, NLR and PNI as important predictors of CPSP after THA in patients with HF, suggesting that preoperative nutritional reserve and immune-inflammatory status may contribute to the postoperative pain recovery process. Unlike traditional pain prediction models that mainly focus on local injury severity and pain-related factors, our findings emphasize the potential role of systemic physiological status in postoperative pain recovery. For patients with HF, surgical intervention represents not only local tissue injury but also a substantial systemic stress event. Therefore, the availability of sufficient nutritional reserve and immune regulatory capacity may influence the ability of patients to transition from acute postoperative pain toward a normal recovery trajectory. GNRI is a comprehensive indicator used to assess nutritional risk in older adults, incorporating serum albumin levels, body weight, and age-related factors to reflect long-term nutritional status and disease-related vulnerability. In the present study, GNRI was not only retained in the final prediction model but also demonstrated the highest contribution in SHAP analysis (35.4%), suggesting that nutritional status may represent a critical determinant of CPSP development. Previous studies have demonstrated that malnutrition is common among older orthopedic patients and is associated with postoperative infection, delayed wound healing, impaired functional recovery, and increased mortality risk (20). However, the relationship between nutritional status and chronic postoperative pain remains insufficiently investigated. Our findings further suggest that nutritional risk may influence not only conventional postoperative recovery outcomes but also the process of pain persistence. The mechanisms linking malnutrition to CPSP may involve multiple interconnected pathways. First, inadequate nutritional reserve is often accompanied by impaired protein synthesis and abnormal skeletal muscle metabolism. Skeletal muscle serves as an important protein reservoir and plays essential roles in tissue repair, energy metabolism, and immune regulation. Nutritional deficiency may promote muscle protein degradation, reduce patients' ability to maintain functional activity and participate in rehabilitation, and subsequently increase the risk of delayed recovery and persistent pain (28, 29). Second, nutritional status is closely associated with immune function. Insufficient protein and related nutrients may impair immune cell function and inflammatory regulation, leading to a persistent low-grade inflammatory state. Previous studies have shown that chronic inflammation can enhance nociceptive pathway activation and contribute to peripheral and central sensitization, which are important mechanisms underlying pain chronicization (30, 31). In addition to GNRI, PNI was also retained in the final prediction model, suggesting that immune-related nutritional status may provide complementary information for CPSP prediction. PNI integrates serum albumin levels and lymphocyte counts, reflecting both nutritional reserve and immune function. Although its contribution in SHAP analysis was lower than that of GNRI (9.5%), the inclusion of PNI indicates that impaired nutritional and immune status may jointly influence postoperative pain recovery. NLR was also identified as a key predictor in the final model, further supporting the role of inflammatory responses in CPSP development. NLR is an easily accessible systemic inflammatory marker that reflects the balance between innate immune activation and adaptive immune status. HF itself can induce a significant inflammatory response, while THA may further enhance immune activation. In some patients, inflammatory responses may fail to return to physiological homeostasis, and persistent inflammatory burden may subsequently influence postoperative pain recovery (32). From a biological perspective, sustained inflammation may contribute to CPSP through the promotion of peripheral and central sensitization. Activated neutrophils can release multiple pro-inflammatory mediators, including interleukin-6, tumor necrosis factor-alpha, and reactive oxygen species, which may enhance nociceptor excitability and increase pain sensitivity. Meanwhile, inflammatory mediators may promote glial cell activation and alter spinal pain modulation, facilitating the transition from acute postoperative pain to persistent pain (31, 33). Furthermore, persistent inflammation may impair tissue repair and functional recovery, thereby prolonging pain duration. Notably, the simultaneous inclusion of GNRI, NLR and PNI in the optimal prediction model suggests that nutritional status and inflammatory burden may not represent independent risk domains but rather complementary components reflecting patients' overall recovery capacity. Nutritional deficiency may weaken the ability of the body to regulate inflammatory responses, whereas persistent inflammation may promote protein catabolism, muscle depletion, and functional decline, forming a vicious cycle of “nutritional deficiency–inflammatory activation–muscle deterioration–impaired recovery.” This process is consistent with the recently proposed inflammaging–frailty axis, in which age-related chronic inflammation and declining physiological reserve jointly contribute to adverse health outcomes in older adults (34, 35). The present findings further indicate that CPSP risk assessment should not focus solely on pain-related characteristics but should also consider whether patients possess sufficient physiological capacity for tissue repair, functional recovery, and restoration of neurophysiological regulation. GNRI, NLR and PNI are derived from routinely available preoperative clinical data and have advantages including accessibility, low cost, and repeatability, supporting their potential clinical applicability. For patients with low preoperative GNRI or PNI, or elevated NLR, future studies should investigate whether nutritional optimization, inflammatory risk management, and enhanced rehabilitation strategies can improve long-term pain outcomes. In summary, our findings suggest that GNRI, NLR and PNI are not merely indicators of nutritional status and inflammatory burden but may also represent the overall recovery environment influencing CPSP development. Integrating nutritional and inflammatory factors into machine learning-based prediction frameworks may provide a systemic perspective for understanding CPSP mechanisms after THA in patients with HF and offer new evidence for individualized perioperative management.

The present study further demonstrated that CT-derived peri-hip muscle parameters, particularly gluteus medius/minimus muscle density and CSA, provided important predictive value for CPSP after THA in patients with HF. Previous CPSP studies have primarily focused on pain intensity, nerve injury, psychological status, and perioperative factors, whereas preoperative muscle status, an important indicator of physiological reserve and recovery potential, has received relatively limited attention. Our findings suggest that muscle reserve may represent a critical link connecting preoperative physical condition with postoperative pain recovery trajectories. Skeletal muscle not only contributes to locomotion and mechanical function but also plays important roles in metabolic regulation, immune homeostasis, and tissue repair. With increasing recognition of the endocrine function of skeletal muscle and muscle–immune interactions, skeletal muscle is now considered an important regulator of systemic recovery capacity rather than merely a mechanical tissue (36, 37). For patients undergoing major orthopedic surgery, postoperative recovery depends largely on the ability of the body to adapt to surgical stress, and reduced muscle mass or impaired muscle quality may compromise this adaptive capacity, thereby increasing the risk of delayed functional recovery and persistent pain. In the present study, both CSA and muscle density were incorporated to characterize muscle status from two complementary dimensions, reflecting muscle quantity and muscle quality, respectively. Notably, the final model included not only a muscle quantity parameter but also a muscle quality parameter, namely gluteus medius/minimus CSA and muscle density, suggesting that both structural characteristics and tissue quality of skeletal muscle may contribute to CPSP risk. Increasing evidence indicates that relying solely on muscle area to evaluate muscle status has limitations, whereas reduced muscle density, reflecting increased intramuscular fat infiltration, may better capture impaired muscle function and metabolic abnormalities (38). Decreased muscle density is commonly accompanied by increased fat deposition, impaired muscle fiber function, altered energy metabolism, and reduced physical capacity, which may compromise postoperative rehabilitation and increase the likelihood of persistent pain (39, 40). Several potential mechanisms may explain the association between impaired muscle status and CPSP development. First, myosteatosis is closely associated with chronic low-grade inflammation, and abnormal muscle tissue may release various inflammatory mediators that contribute to a systemic pro-inflammatory environment (41). Second, impaired muscle function may reduce the production of myokines and influence inflammatory regulation and tissue repair processes. For example, irisin and other muscle-derived factors have been implicated in metabolic regulation and anti-inflammatory responses, and alterations in these pathways may affect recovery after tissue injury (36, 42). Persistent inflammatory activation may further promote peripheral nociceptor sensitization and abnormal central pain modulation, increasing the susceptibility to transition from acute postoperative pain to chronic pain (43). The inclusion of gluteus medius/minimus parameters in the final prediction model also has anatomical and functional relevance. The gluteus medius and gluteus minimus muscles are essential for maintaining pelvic stability, controlling hip abduction, and preserving normal gait patterns. In patients with HF, early postoperative mobilization and functional rehabilitation rely heavily on these muscle groups, and muscle atrophy or fatty degeneration may impair weight-bearing capacity, alter gait mechanics, and limit rehabilitation participation (44). Reduced physical activity may not only delay recovery but also increase pain-related attention and avoidance behaviors, thereby creating a reciprocal cycle between persistent pain and functional decline. Furthermore, the relationship between muscle status and pain recovery may be dynamic. Insufficient preoperative muscle reserve may increase the risk of persistent postoperative pain, whereas prolonged pain may subsequently reduce physical activity and accelerate muscle functional deterioration. Therefore, muscle abnormalities may represent not only a risk marker for CPSP but also a potential contributor to its persistence. This feedback process of “low muscle reserve–impaired functional recovery–reduced activity–persistent pain” may partly explain why some patients with HF experience prolonged recovery after surgery (45). From a clinical perspective, our findings support the incorporation of opportunistic CT-based muscle assessment into predischarge risk stratification for patients with HF. Since these patients commonly undergo preoperative CT examinations, obtaining muscle CSA and density measurements from existing imaging data does not impose additional examination burden and therefore has good clinical feasibility. In the future, combining CT-derived muscle phenotyping with nutritional optimization, rehabilitation strategies, and preventive pain management approaches may facilitate the identification of high-risk patients and provide new opportunities for individualized perioperative care.

The present study identified preoperative anxiety as one of the important psychological features for predicting CPSP after THA in patients with HF. This finding further supports the current understanding of CPSP development, indicating that chronic pain is not determined solely by the extent of tissue injury but is influenced by multiple interacting factors, including pain perception, emotional status, and central nervous system regulation. For patients with HF, the sudden traumatic event, reduced mobility, uncertainty regarding surgical outcomes, and concerns about future functional recovery may induce substantial psychological stress, and this preoperative psychological state may subsequently influence postoperative pain recovery trajectories. Previous studies have demonstrated that preoperative anxiety is closely associated with persistent pain after various surgical procedures (46). Anxiety may influence pain processing by altering cognitive responses to nociceptive stimuli, leading patients to exhibit greater attention toward pain signals and stronger threat appraisal, thereby amplifying pain perception. Furthermore, patients with anxiety often exhibit more negative pain expectations and a greater tendency toward pain catastrophizing, making them more likely to interpret pain as a threatening signal and underestimate their recovery capacity. This maladaptive pain cognition pattern may subsequently affect postoperative rehabilitation behaviors and increase the likelihood of persistent pain. From a neurobiological perspective, anxiety is not merely an emotional response but involves complex neural regulatory processes. Persistent anxiety may impair the function of descending pain modulation systems and reduce the endogenous inhibitory capacity for nociceptive stimuli, making normal tissue repair-related postoperative pain more likely to be amplified and maintained (47). In addition, emotional processing regions and pain-related brain regions, including the prefrontal cortex, insula, anterior cingulate cortex, and amygdala, are closely interconnected. Anxiety may alter information integration among these regions, enhance pain-related emotional responses, and facilitate the transition from acute pain to chronic pain (48). Psychological factors may also influence postoperative recovery through behavioral pathways. According to the fear-avoidance model, excessive attention to pain and fear of pain may lead patients to reduce physical activity and participate less in rehabilitation training, resulting in impaired muscle function, restricted joint mobility, and delayed functional recovery (45). For patients with HF, early mobilization and functional rehabilitation are essential for regaining independent living ability, whereas anxiety-related activity avoidance may further aggravate functional impairment and contribute to a reciprocal interaction among pain, reduced activity, and poor recovery. Furthermore, psychological stress may interact with inflammatory responses. Chronic anxiety may influence stress responses through the hypothalamic–pituitary–adrenal axis and alter immune-inflammatory regulation. Previous studies have suggested that increased psychological stress may be accompanied by elevated pro-inflammatory mediators, while inflammatory environments may further affect neurotransmitter balance and pain sensitivity, creating a process in which psychological and biological factors jointly contribute to pain persistence (49). Notably, the present study did not evaluate psychological factors as isolated variables but integrated preoperative anxiety with nutritional indicators, inflammatory markers, and CT-derived muscle phenotypes within the ML framework. This finding suggests that anxiety may reflect not only emotional status but also patients' adaptive capacity when facing disease, surgery, and rehabilitation. Compared with relying solely on preoperative pain scores, psychological factors may provide additional information regarding pain susceptibility and help identify high-risk patients who cannot be recognized by conventional clinical indicators. From a clinical perspective, preoperative anxiety is relatively easy to identify and potentially modifiable, suggesting its value in perioperative management of patients with HF. For patients with significant anxiety symptoms, additional interventions beyond conventional analgesic strategies, including preoperative education, pain cognition-based interventions, psychological support, and individualized rehabilitation plans, may help reduce pain-related fear and activity avoidance and promote postoperative recovery. Future studies are warranted to determine whether psychological interventions can modify CPSP trajectories and to explore potential interactions between psychological status, muscle characteristics, and inflammatory burden. In summary, the present study indicates that preoperative anxiety represents an important psychological susceptibility factor for CPSP after THA in patients with HF, with potential mechanisms involving altered pain cognition, impaired descending pain modulation, neuroinflammatory regulation, and changes in rehabilitation behaviors. Incorporating psychological characteristics into prediction models integrating imaging-derived and biological markers may further improve CPSP risk stratification and provide new evidence for individualized pain prevention strategies.

Age was also identified as an important predictor in the present model, indicating that aging-related physiological decline may contribute to susceptibility to CPSP after THA. Older patients often exhibit reduced tissue repair capacity, impaired neuromuscular function, decreased physiological reserve, and increased vulnerability to systemic stress. These age-related changes may compromise postoperative recovery and prolong the transition from acute surgical pain to persistent pain. Therefore, age may serve not only as a demographic characteristic but also as a surrogate marker reflecting the overall recovery capacity of older patients.

The present study applied ML approaches to integrate clinical characteristics, psychological factors, nutrition- and inflammation-related indicators, and CT-derived muscle phenotypes to develop and validate a prediction model for CPSP after THA in patients with HF. Compared with traditional prediction models constructed based on univariable analyses or linear assumptions, the primary value of this approach was not simply to compare the performance of different algorithms, but rather to characterize the complex biological features associated with postoperative pain recovery through multidimensional data integration. The development of CPSP involves multiple interacting domains, including tissue injury, inflammatory responses, neural regulation, psychological status, and physiological reserve. Therefore, ML methods capable of handling heterogeneous variables and capturing potential nonlinear relationships may provide a novel framework for predicting such multifactorial clinical outcomes. Conventional clinical prediction models generally rely on a limited number of prespecified variables and assume linear associations between predictors and outcomes. However, in real-world clinical settings, postoperative pain recovery is rarely determined by a single factor. For example, impaired nutritional status may simultaneously affect muscle reserve, inflammatory regulation, and rehabilitation capacity, while psychological factors may modify pain perception and behavioral responses. These complex interactions may not be fully captured by conventional statistical approaches. Although the present model integrated clinical, nutritional, inflammatory, and CT-derived muscle indicators, its discrimination in the temporal test cohort was comparable to that of conventional logistic regression. Therefore, our findings should not be interpreted as demonstrating the superiority of SVM over conventional prediction approaches. In the present study, the combination of LASSO and Boruta algorithms was used to identify predictors with stable predictive value from different perspectives, followed by the development of multiple ML models to improve model robustness and reliability. The final model incorporated GNRI, gluteus medius/minimus muscle density, gluteus medius/minimus CSA, preoperative anxiety, NLR, age, and PNI. These variables represent different dimensions of patient status, including nutritional reserve, muscle characteristics, inflammatory burden, psychological susceptibility, and baseline physiological condition, thereby forming a multidimensional risk phenotype. This finding suggests that CPSP risk assessment may need to move beyond focusing solely on pain-related factors toward a comprehensive evaluation of patients' preoperative “recovery capacity.” This concept is consistent with the increasing emphasis in perioperative medicine on physiological reserve and recovery potential, indicating that long-term postoperative outcomes are determined not only by surgical procedures but also by patients' ability to adapt to physiological stress (16). Furthermore, this study applied SHAP analysis to interpret the optimal model, thereby improving the transparency of the machine learning framework. One of the major challenges in applying ML models in medicine is their potential “black-box” nature, in which models can generate risk predictions but provide limited insight into the underlying decision-making process. Through SHAP analysis, the present study quantified the contribution of individual predictors to model outputs and illustrated the direction of their associations with CPSP risk. The results showed that GNRI contributed most substantially to the model, followed by gluteus medius/minimus muscle density and NLR, further supporting the important roles of nutritional status, muscle quality, and inflammatory burden in CPSP development. Compared with providing risk probabilities alone, model interpretability analysis may help clinicians understand individual risk profiles and provide potential guidance for targeted interventions. Another important strength of this study was the use of an independent temporal test set for model validation. The generalizability of machine learning models in clinical applications has received increasing attention. Validation based solely on random training and validation splits may overestimate model performance because patients and healthcare processes are derived from similar sources. In contrast, validation using consecutively enrolled patients from a subsequent time period more closely reflects real-world clinical implementation. In the present study, independent temporal validation demonstrated that the SVM model maintained favorable discrimination and calibration performance, suggesting good model stability and potential generalizability.

This study has several strengths. First, compared with previous CPSP prediction studies that primarily relied on age, preoperative pain, and psychological factors, the present study incorporated a broader range of candidate predictors, including psychological characteristics, nutrition- and inflammation-related indicators, and CT-derived muscle phenotypes. By evaluating patients' preoperative recovery reserve from multiple dimensions, this approach improved the ability of the model to characterize complex clinical conditions. Second, this study applied machine learning strategies for feature selection and model development and further incorporated SHAP analysis to quantify the contribution of individual variables to model predictions, thereby improving model transparency and clinical interpretability. The independent temporal test set validation further strengthened the stability of the model. However, several limitations should be acknowledged. First, this was a single-center cohort study. Although an independent temporal test set was used for validation, the sample size of the test cohort remained relatively limited, and external validation across multiple centers is still lacking. Although the EPV analysis exceeded the minimum requirement for model development, which may reduce the risk of overfitting and improve the reliability of parameter estimation, further validation in larger prospective multicenter cohorts is required to confirm the generalizability of the model. Second, although SHAP analysis improved the interpretability of the machine learning model, the model outputs primarily represent statistical associations between predictors and CPSP risk and cannot directly establish causal relationships between individual predictors and CPSP development. Therefore, muscle-related parameters, nutrition- and inflammation-related indicators, and psychological factors identified in this study should be considered risk markers rather than definitive causal factors. Future studies integrating mechanistic investigations and interventional trials are needed to further clarify their roles. Third, this study mainly analyzed perioperative information obtained from hospital admission to discharge. Although all patients received standardized analgesic guidance, rehabilitation recommendations, and follow-up management, individual patient needs, treatment preferences, rehabilitation adherence, and lifestyle-related factors may also influence pain recovery trajectories and were not fully captured in the model. Future studies should incorporate longitudinal pain trajectories, functional recovery outcomes, implant survival, and patient-reported outcomes to provide a more comprehensive assessment of postoperative recovery. Fourth, although contralateral muscle measurements were used to reduce the influence of local injury, pain, and immobilization on the fracture side, the contralateral muscle status may still have been affected by pre-existing bilateral sarcopenia, frailty, or chronic disuse in this older population. Therefore, these measurements may not fully represent the patients' pre-fracture muscle reserve. Finally, CPSP itself contains a subjective component, as pain assessment may be influenced by psychological status, individual pain thresholds, and variations in pain expression. Therefore, integrating objective functional measurements with multidimensional outcome assessments may further enhance the clinical applicability of future prediction models.

## 5 Conclusion

This study developed and validated a machine learning-based prediction model for CPSP after THA in patients with HF. By integrating preoperative clinical characteristics, psychological factors, nutrition- and inflammation-related indicators, and CT-derived muscle phenotypes, the model effectively identified patients at high risk of CPSP. GNRI, preoperative anxiety, gluteus medius/minimus muscle density, gluteus medius/minimus CSA, age, NLR, and PNI were identified as important predictors, highlighting the contribution of physiological reserve, muscle status, nutritional condition, inflammatory burden, and psychological susceptibility to CPSP development. The interpretable machine-learning model provides a potential approach for predischarge risk stratification and individualized postdischarge management. Future multicenter external validation and prospective intervention studies are needed to further evaluate its clinical applicability.

## Data Availability

The raw data supporting the conclusions of this article will be made available by the authors, without undue reservation.
