# Supplementary material for: Nutritional status, inflammatory burden, and CT-derived muscle phenotypes for predicting chronic postsurgical pain after total hip arthroplasty: an explainable machine learning study
Source: Front Nutr. 2026 Oct 20;13:1958865. doi: 10.3389/fnut.2026.1958865 (PMC13727434; doi:10.3389/fnut.2026.1958865)
Supplement: Supplementary file 1 [file Table_1.docx]

Supplementary Table 1 Baseline characteristics of patients in the development and test cohorts

| Variable | Development cohort (n=529) | Test cohort (n=352) | P value |
| --- | --- | --- | --- |
| Preoperative NRS (score) | 7.00 (6.00, 8.00) | 7.00 (6.00, 8.00) | 0.062 |
| Postoperative 48-h NRS (score) | 4.00 (3.00, 4.00) | 3.00 (2.00, 4.00) | 0.099 |
| CPSP (Yes/No) | 137/392 | 90/262 | 0.913 |
| Age (years) | 67.00 (63.00, 71.00) | 68.00 (64.00, 71.00) | 0.494 |
| Sex (Male/Female) | 190/339 | 136/216 | 0.413 |
| BMI (kg/m2) | 23.85 (20.55, 27.11) | 23.42 (20.91, 26.91) | 0.519 |
| Education level (≥Secondary/<Secondary) | 209/320 | 119/233 | 0.086 |
| Preoperative long-term analgesic use (Yes/No) | 173/356 | 120/232 | 0.668 |
| Smoking history (Yes/No) | 194/335 | 117/235 | 0.296 |
| Alcohol history (Yes/No) | 164/365 | 93/259 | 0.143 |
| Hypertension (Yes/No) | 249/280 | 155/197 | 0.376 |
| Diabetes (Yes/No) | 127/402 | 90/262 | 0.598 |
| Heart disease (Yes/No) | 132/397 | 105/247 | 0.110 |
| History of cerebral infarction (Yes/No) | 102/427 | 81/271 | 0.181 |
| Preoperative anxiety (Yes/No) | 148/381 | 83/269 | 0.146 |
| Preoperative depression (Yes/No) | 100/429 | 74/278 | 0.439 |
| Pain catastrophizing (Yes/No) | 129/400 | 98/254 | 0.251 |
| Gluteus maximus CSA (cm2) | 30.45 (25.69, 34.92) | 30.74 (26.63, 34.29) | 0.961 |
| Gluteus maximus HU (HU) | 23.66 (20.01, 28.53) | 24.05 (20.86, 28.08) | 0.195 |
| Rectus femoris CSA (cm2) | 3.74 (2.76, 4.68) | 3.85 (2.98, 4.77) | 0.278 |
| Rectus femoris HU (HU) | 41.31 (37.42, 45.64) | 41.75 (37.19, 44.65) | 0.243 |
| Gluteus medius/minimus CSA (cm2) | 30.56 (26.11, 35.09) | 29.21 (24.58, 35.08) | 0.096 |
| Gluteus medius/minimus HU (HU) | 31.73 (28.04, 35.95) | 31.05 (27.24, 34.64) | 0.095 |
| ASA physical status (III–IV/I–II) | 251/278 | 159/193 | 0.507 |
| General anesthesia (Yes/No) | 358/171 | 222/130 | 0.158 |
| Surgery delay >48 h (Yes/No) | 339/190 | 203/149 | 0.055 |
| High-energy injury (Yes/No) | 146/383 | 103/249 | 0.592 |
| Surgery duration (min) | 65.00 (60.00, 75.00) | 70.00 (55.75, 75.00) | 0.489 |
| Operative side (Right/Left) | 262/267 | 183/169 | 0.474 |
| Intraoperative blood loss (mL) | 400.00 (360.00, 440.00) | 400.00 (350.00, 430.00) | 0.094 |
| Transfusion (Yes/No) | 126/403 | 71/281 | 0.203 |
| CAR | 1.55 (1.07, 2.11) | 1.62 (0.98, 2.21) | 0.933 |
| GNRI | 92.58 (89.93, 95.14) | 92.37 (90.26, 94.44) | 0.335 |
| PNI | 41.60 (39.30, 44.25) | 42.04 (39.42, 44.91) | 0.441 |
| NLR | 6.78 (4.72, 8.76) | 6.50 (4.47, 8.63) | 0.380 |
| SII | 1067.53 (667.48, 1755.40) | 1044.51 (689.67, 2080.97) | 0.544 |

Abbreviations: NRS, Numeric Rating Scale; CPSP, chronic postsurgical pain; BMI, body mass index; CSA, cross-sectional area; HU, Hounsfield units; ASA, American Society of Anesthesiologists; CAR, C-reactive protein-to-albumin ratio; GNRI, Geriatric Nutritional Risk Index; PNI, Prognostic Nutritional Index; NLR, neutrophil-to-lymphocyte ratio; SII, Systemic Immune-Inflammation Index.

Supplementary Table 2 Intra-rater and inter-rater reliability of CT-derived muscle parameter measurements

| Parameter | Intra-rater ICC (95% CI) | Inter-rater ICC (95% CI) |
| --- | --- | --- |
| Gluteus maximus CSA | 0.913 (0.784–0.966) | 0.887 (0.730–0.954) |
| Gluteus maximus HU | 0.911 (0.712–0.968) | 0.859 (0.633–0.945) |
| Rectus femoris CSA | 0.983 (0.958–0.993) | 0.966 (0.918–0.986) |
| Rectus femoris HU | 0.900 (0.766–0.959) | 0.871 (0.707–0.946) |
| Gluteus medius/minimus CSA | 0.911 (0.792–0.964) | 0.893 (0.749–0.956) |
| Gluteus medius/minimus HU | 0.865 (0.691–0.945) | 0.850 (0.660–0.938) |

Abbreviations: ICC, intraclass correlation coefficient; CI, confidence interval; CT, computed tomography; CSA, cross-sectional area; HU, Hounsfield units.

Supplementary Table 3 Performance Comparison of SVM and Logistic Regression in the Independent Temporal Test Cohort

| Model | AUC | AUPRC | Brier score | Accuracy | Sensitivity | Specificity | PPV | NPV | F1-score |
| --- | --- | --- | --- | --- | --- | --- | --- | --- | --- |
| Logistic Regression | 0.854 | 0.757 | 0.122 | 0.813 | 0.678 | 0.859 | 0.622 | 0.886 | 0.649 |
| SVM | 0.854 | 0.754 | 0.125 | 0.770 | 0.789 | 0.763 | 0.534 | 0.913 | 0.637 |

Abbreviations: SVM, support vector machine; LR, logistic regression; AUC, area under the receiver operating characteristic curve; AUPRC, area under the precision–recall curve; PPV, positive predictive value; NPV, negative predictive value.
